# Unsuspected Involvement of Spinal Cord in Alzheimer Disease

**DOI:** 10.3389/fncel.2020.00006

**Published:** 2020-01-30

**Authors:** Roberta Maria Lorenzi, Fulvia Palesi, Gloria Castellazzi, Paolo Vitali, Nicoletta Anzalone, Sara Bernini, Matteo Cotta Ramusino, Elena Sinforiani, Giuseppe Micieli, Alfredo Costa, Egidio D’Angelo, Claudia A. M. Gandini Wheeler-Kingshott

**Affiliations:** ^1^Department of Brain and Behavioral Sciences, University of Pavia, Pavia, Italy; ^2^Neuroradiology Unit, Brain MRI 3T Research Center, IRCCS Mondino Foundation, Pavia, Italy; ^3^Department of Electrical, Computer and Biomedical Engineering, University of Pavia, Pavia, Italy; ^4^Queen Square MS Centre, Department of Neuroinflammation, UCL Queen Square Institute of Neurology, Faculty of Brain Sciences, University College London, London, United Kingdom; ^5^Universita Vita e Salute, S Raffaele Hospital Milan, Milan, Italy; ^6^Laboratory of Neuropsychology, IRCCS Mondino Foundation, Pavia, Italy; ^7^Unit of Behavioral Neurology, IRCCS Mondino Foundation, Pavia, Italy; ^8^Department of Emergency Neurology, IRCCS Mondino Foundation, Pavia, Italy; ^9^Brain Connectivity Center (BCC), IRCCS Mondino Foundation, Pavia, Italy; ^10^Brain MRI 3T Research Center, IRCCS Mondino Foundation, Pavia, Italy

**Keywords:** dementia—Alzheimer’s disease, cross-sectional area (CSA), brain atrophy, spinal cord atrophy, spinal cord toolbox, Alzheimer’s diagnosis, dementia biomarker, sensorymotor function impairment

## Abstract

**Objective**: Brain atrophy is an established biomarker for dementia, yet spinal cord involvement has not been investigated to date. As the spinal cord is relaying sensorimotor control signals from the cortex to the peripheral nervous system and vice-versa, it is indeed a very interesting question to assess whether it is affected by atrophy due to a disease that is known for its involvement of cognitive domains first and foremost, with motor symptoms being clinically assessed too. We, therefore, hypothesize that in Alzheimer’s disease (AD), severe atrophy can affect the spinal cord too and that spinal cord atrophy is indeed an important *in vivo* imaging biomarker contributing to understanding neurodegeneration associated with dementia.

**Methods**: 3DT1 images of 31 AD and 35 healthy control (HC) subjects were processed to calculate volume of brain structures and cross-sectional area (CSA) and volume (CSV) of the cervical cord [per vertebra as well as the C2-C3 pair (CSA23 and CSV23)]. Correlated features (*ρ* > 0.7) were removed, and the best subset identified for patients’ classification with the Random Forest algorithm. General linear model regression was used to find significant differences between groups (*p* ≤ 0.05). Linear regression was implemented to assess the explained variance of the Mini-Mental State Examination (MMSE) score as a dependent variable with the best features as predictors.

**Results**: Spinal cord features were significantly reduced in AD, independently of brain volumes. Patients classification reached 76% accuracy when including CSA23 together with volumes of hippocampi, left amygdala, white and gray matter, with 74% sensitivity and 78% specificity. CSA23 alone explained 13% of MMSE variance.

**Discussion**: Our findings reveal that C2-C3 spinal cord atrophy contributes to discriminate AD from HC, together with more established features. The results show that CSA23, calculated from the same 3DT1 scan as all other brain volumes (including right and left hippocampi), has a considerable weight in classification tasks warranting further investigations. Together with recent studies revealing that AD atrophy is spread beyond the temporal lobes, our result adds the spinal cord to a number of unsuspected regions involved in the disease. Interestingly, spinal cord atrophy explains also cognitive scores, which could significantly impact how we model sensorimotor control in degenerative diseases with a primary cognitive domain involvement. Prospective studies should be purposely designed to understand the mechanisms of atrophy and the role of the spinal cord in AD.

## Introduction

Dementia is one of the most debilitating cognitive neurodegenerative disorders affecting the central nervous system in elderly people and having a significant impact on daily life activities. With an aging population, the incidence of dementia is growing and the consequences on society are huge. Clinically, several forms of dementia-like diseases that differently impair multiple cognitive and behavioral domains are defined. Alzheimer’s disease (AD) is the most common cause of dementia and it is responsible for 60–80% of cases worldwide (Kumar and Singh, 2015). What is the effect of neurodegeneration on sensorimotor control is an interesting question because it is believed to be highly relevant also for understanding cognitive functions. As the spinal cord is relaying sensorimotor control signals from the cortex to the peripheral nervous system and vice versa, it is indeed important to assess whether it is affected by atrophy in a disease that is known for its involvement of cognitive domains. Recent indications suggest that there is definitely a sensorimotor network rewiring and that the motor system may even be affected before cognitive functions in AD (Agosta et al., 2010; Salustri et al., 2013; Castellazzi et al., 2014; Albers et al., 2015; Fu et al., 2018). Clinical symptoms of early AD include, amongst others, fine motor impairment, with for example worsening of writing abilities. Post-mortem histopathology has indicated that phosphorylated tau tangles are present in high proportion in the cervical spinal cord of AD cases compared to healthy subjects (Dugger et al., 2013). This is also supported by studies in different animal models of AD where pathological changes are demonstrated in the spinal cord as well as the brain (Yuan et al., 2013; Chu et al., 2017). Therefore, it is important to understand first of all whether the spinal cord plays a part in this disease and to understand how significant its involvement is.

AD is associated with an extracellular deposit of β-amyloid plaques in the brain and cerebral vessels, but also to the presence of intracellular neurofibrillary tangles, which appear like paired helical filaments with hyperphosphorylated tau proteins. Tau tangles have been identified as the cause of cortical neurons’ degeneration while amyloid-β (Aβ) oligomers have an important role in synaptic impairment, hence Aβ plaques deposition is suggested to raise later during the AD progression (Song et al., 2014; Šimić et al., 2016). This neuronal degeneration explained by pathophysiology leads to macroscopic atrophy of specific brain structures, such as the hippocampi and the medial temporal lobes (Scher et al., 2011), which can be detected using Magnetic Resonance Imaging (MRI) techniques. Indeed, several MRI studies have demonstrated significant atrophy of white matter (WM), gray matter (GM) and specific brain structures such as the hippocampi, thalami, and amygdalae in AD patients suggesting that these structures are informative in identifying dementia disorders (Stonnington et al., 2010; Pini et al., 2016). The hippocampi have been proposed as *in vivo* non-invasive imaging biomarkers of AD while other structures may be useful in distinguishing between different subtypes of dementia (Palesi et al., 2018). Only far and few old studies have looked at the spinal cord in AD, from a postmortem histochemical analysis and with reference to the autonomic system, but results were never reproduced or follow through as they focused on tau pathology, which was only sporadically reported (Engelhardt and Laks, 2016).

Recently, numerous MRI investigations have tried to identify new *in vivo* biomarkers for dementia to understand mechanisms of AD, to have better tools for assessing new therapies and predicting the clinical evolution of prodromic stages of dementia. Optical Coherence Tomography studies, for example, have been used to demonstrate that retinal ganglion cell degeneration can be associated with early stages of AD. Also, structures like the cerebellum, not classically associated with AD, have been found to be altered in imaging studies of dementia (Castellazzi et al., 2014), with atrophy of the anterior cerebellum—known for its motor control—being present even in the prodromic stages of mild cognitive impairment (MCI; Toniolo et al., 2018). Recent work has also looked at graph theory metrics to distinguish patterns of AD, identifying potentially different subtypes (Ferreira et al., 2019), although focusing on cortical and deep gray matter areas, without including the cerebellum and the spinal cord. Studies of other diseases associated with neurodegeneration, such as multiple sclerosis (Liu et al., 2015), amyotrophic lateral sclerosis (Antonescu et al., 2018), and spinal cord injury (Grussu et al., 2017), have revealed that atrophy of the spinal cord is indicative of widespread alterations of the central nervous system and might be considered as a relevant imaging biomarker in a wider range of neurodegenerative diseases. Nevertheless, this kind of alteration has never been investigated and reported in dementia patients *in vivo* and MRI offers such a possibility with existing datasets covering brain and spine. Hence, the main aim of the present retrospective work was in the first instance to assess whether spinal cord volume is reduced in AD patients compared to healthy controls (HC), hypothesizing that the neurodegeneration typical of AD spreads to all components of the central nervous system; we achieved this by comparing a number of spinal cord features from an existing structural dataset between AD and HC. This information is very important for our understanding of how a neurodegenerative disease like AD has implications beyond the known brain atrophy: this could also have a significant impact on future modeling of brain networks. Furthermore, in case of a positive outcome, it is important to quantify the role of spinal cord features in distinguishing between AD and HC to drive the design of future studies; for this we implemented a machine learning approach for features selection, that is increasingly applied to improve diagnostic accuracy by quantitative imaging (Dauwan et al., 2016; Mirzaei et al., 2016). Finally, we quantified the contribution of spinal cord atrophy to explain the variance of clinical scores for determining its clinical relevance.

## Materials and Methods

### Subjects

A total of 66 subjects including 31 AD patients (age (73 ± 7) years, 12 females (F), Mini-Mental State Examination (MMSE) = 16 ± 6) and 35 HC (age (69 ± 10) years, 17 F, MMSE = 28 ± 1), as a reference group, were analyzed. Seven subjects (three HC and three AD) were excluded from the study due to post-processing issues, hence the final dataset comprised 32 HC and 28 AD.

Inclusion criteria for patients were: clinical diagnosis of dementia on the basis of the Diagnostic and Statistical Manual of Mental Disorders (DSM-5) criteria (American Psychiatric Association, 2013), MMSE score (Folstein et al., 1975) below 24 and age above 60 years. Exclusion criteria comprised the presence of at least one of the following: epilepsy or isolated seizures, major psychiatric disorders over the previous 12 months, pharmacologically treated delirium or hallucinations, ongoing alcoholic abuse, acute ischemic or hemorrhagic stroke, known intracranial lesions, and systemic causes of subacute cognitive impairment (Geschwind et al., 2009). Diagnosis of AD was made according to the criteria of the National Institute of Neurological and Communicative Disorders and Stroke and AD and Related Disorders Association (NIA-AA 1011) workgroup (McKhann et al., 2011). HC was enrolled on a voluntary basis among subjects with MMSE score above 27 and attending a local third age university (University of Pavia, Information Technology course) or included in a program on healthy aging (Fondazione Golgi, Abbiategrasso, Italy).

The study was accomplished in accordance with the Declaration of Helsinki and with the approbation of the local ethics committee of the IRCCS Mondino Foundation, upon signature of the written informed consent by the subjects.

### MRI Acquisition

High-resolution 3D T1-weighted (3DT1-w) MR images were acquired using a Siemens MAGNETOM Skyra3T (Siemens AG, Erlangen, Germany) with software version NUMARIS/4 (syngo MR D13C version) and a receiving head-coil with 32 channels.

Scan parameters were (Palesi et al., 2018): TR = 2300 ms, TE = 2.95 ms, TI = 900 ms, flip angle = 9°, field of view (FOV) = 269 × 252 mm^2^, acquisition matrix = 256 × 240, in-plane resolution = 1.05 × 1.05 mm^2^, slice thickness = 1.2 mm, and 176 sagittal slices. The FOV, in feet-to-head direction, was set to cover the entire brain and cervical cord up to the C5 vertebra in all subjects.

### Spinal Cord Analysis

For each subject, the 3DT1-w volume (the same used normally for brain atrophy measurements—see below) was resized removing the brain and centered on the spine. Once a single volume of interest (VOI) comprising the same spinal cord regions for each 3DT1-w was defined (matrix = 176 × 240 × 96 voxels), the process was automatized for the whole dataset. The resized 3DT1-w volumes were analyzed with the Spinal Cord Toolbox^1^, an open-source software specifically developed to elaborate spinal cord images, to extract features of the C1-C5 vertebrae.

**Figure 1:**
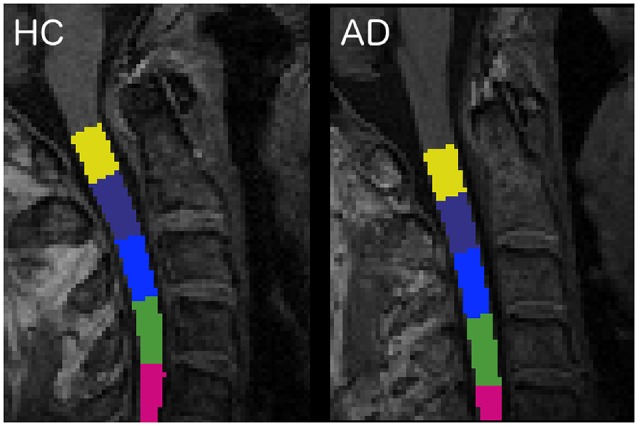
Labeled vertebrae in two randomly chosen subjects: an healthy control (HC) subject on the left and an Alzheimer’s disease (AD) patient on the right (slice *n* = 96, sagittal plane). Each color represents a different vertebra from C1 (yellow) to C5 (fuchsia).

The spinal cord was segmented with the *propseg* algorithm (Yiannakas et al., 2016), which is fully automated, and, after manual initialization, was automatically labeled (Ullmann et al., 2014) to identify all vertebrae separately (Dupont et al., 2017; Figure 1).

Mean cross-sectional area (CSA) and volume (CSV) were calculated for each vertebra and for the C2-C3 pair (CSA23 and CSV23), given the better sensitivity of this combined level to disease severity (Coulon et al., 2002; Liu et al., 2015; Prados et al., 2016; De Leener et al., 2017b). CSA is computed by counting pixels in each slice and then geometrically adjusting it multiplying by the angle (in degrees) between the spinal cord centerline and the inferior-superior direction. CSV, indeed, is computed by counting pixels and multiplying by slice thickness.

### Brain Atrophy Analysis

**Figure 2:**
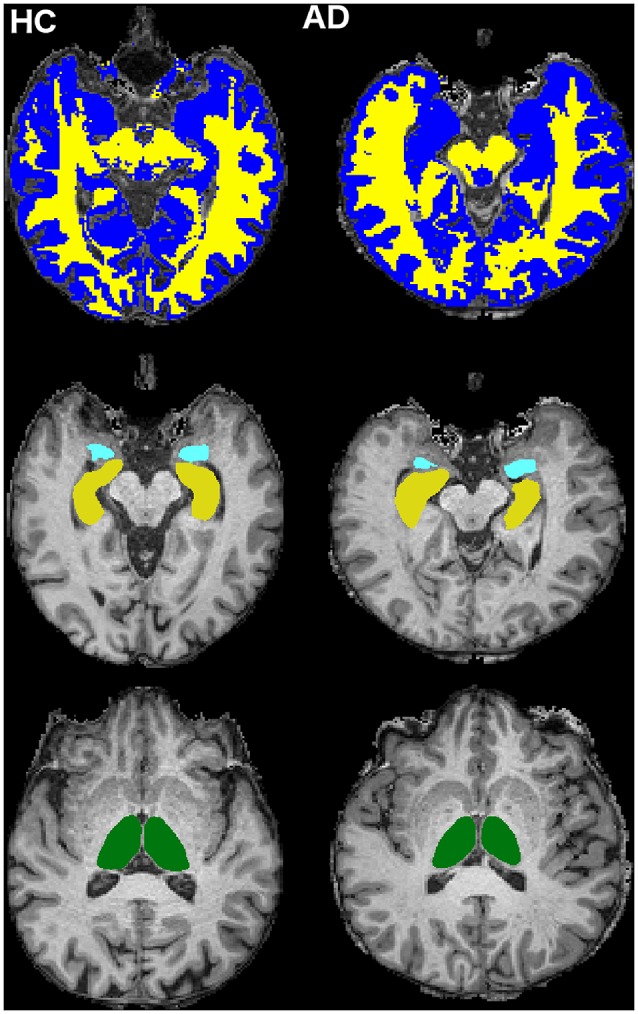
Cerebral tissue segmentation in two randomly chosen subjects: HC subject on the left and AD patient on the right. Top row: white matter (WM; yellow) and gray matter (GM; blue) segmentation (slice *n* = 126, transverse plane). Middle row: hippocampi (yellow) and amygdalae (light blue) segmentation (slice *n* = 123, transverse plane). Bottom row: thalami (green) segmentation (slice *n* = 132, transverse plane).

The 3DT1-w images were also segmented into WM, GM and cerebrospinal fluid (CSF) using SPM12^2^ (Penny et al., 2007), while left (L) and right (R) hippocampi (LHip and RHip), thalami (LThal and RThal) and amygdalae (LAmy and RAmy) were segmented using FIRST (FSL; Patenaude et al., 2011; Figure 2)^3^.

WM, GM, and all other brain structures volumes were calculated in mm^3^. Total intracranial volume, as the sum of WM, GM and CSF, was also calculated to account for different brain sizes.

### Classification of AD and Feature Selection Analysis

Classification between AD and HC was performed using a machine learning approach implemented in Orange^4^.

A total of 22 features were extracted from the above MRI morphometric analysis. Given the large number of parameters extracted compared to the sample size of our AD and HC groups, a feature reduction approach was adopted in order to control for overfitting issues. The Spearman correlation coefficient (Spearman, 1904) was obtained in Matlab between pairs of all calculated metrics. When pairs of metrics had a correlation coefficient greater than 0.7, one metric was kept while the other was eliminated.

Ranking was implemented with the ReliefF algorithm (Urbanowicz et al., 2018) on the uncorrelated features to identify the best subset able to classify AD from HC, and particularly to investigate the contribution of spinal cord metrics to the task. In order to identify a unique subset of features, 30% of instances were employed for ranking. Data were normalized by span to avoid polarization of the results due to the different scales of features, as for WM compared to CSA. The remaining 70% of instances was further divided into 70% for the Random Forest algorithm application and 30% to test its classification accuracy [CA = (True Positive + True Negative)/(True Positive + True Negative + False Positive + False Negative)], sensitivity [Sens = True Positive/(True Positive + False negative)] and specificity [Spec = True Negative/(True Negative + False Positive)], using the previously-identified best features.

Among several machine learning algorithms, RF was selected for its robustness against a reduced number of input features and the capacity to weight features runtime, providing features relevant in a classification task (Breiman, 2001; Goel and Abhilasha, 2017). The Receiving Operating Characteristics (ROC) curve was then obtained to visually discriminate between AD and HC and the Area Under the Curve was also calculated to quantify the overall ability of RF to discriminate between AD and HC.

### Statistical Analysis

Statistical tests were performed using the Statistical Package for Social Sciences (SPSS) software, version 21 (IBM, Armonk, New York, NY, USA). All continuous data were tested for normality using a Shapiro–Wilk test (Shapiro and Wilk, 1965). Age and MMSE were compared between AD and HC using a two-tailed Kruskal–Wallis test (Kruskal and Wallis, 1952) while gender was compared using a chi-squared test (Pearson, 1900). A multivariate regression model with gender, age and total intracranial volume as covariates was used to compare all morphometric metrics between AD and HC. Two-sided *p* < 0.05 was considered statistically significant.

Furthermore, to assess the power of the best features in explaining the variance of the MMSE, a linear regression model was implemented using the MMSE score as the dependent variable and the best features as predictors. These independent features were used in two ways: (i) each predictor was used alone to determine its specific contribution to MMSE; (ii) all features were used in a backward approach to identify which of them explained the greatest percentage of MMSE variance. A threshold of *p* < 0.01 (two-tailed) was considered statistically significant.

## Results

### Subjects

Population demographics and neuropsychological scores are reported in Table 1. Significant differences were found in MMSE between HC and AD patients.

**Table 1:** Subjects’ demographic and neuropsychological data.

	HC (*n* = 32) mean (SD)	AD (*n* = 28) mean (SD)	*p*-value
Age (years)	69.4 (9.6)	73.0 (6.4)	0.138
Gender [Male (%)]	51.4	56.2	0.800
MMSE	28.5 (0.2)	16.0 (1.1)	<0.001*

Gender is expressed in Male % and compared with a Chi-square test. Age and MMSE are expressed as mean (SD) and compared with a Kruskal–Wallis test. Significance was set to *p* = 0.05. *Refers to statistically significant comparisons.

### Morphometric Changes in AD Patients

All results are reported in Tables s 2, 3. AD patients compared to HC showed atrophy in all brain structures. Moreover, all patients for all investigated spinal cord segments showed reduced CSA at all vertebral levels, while CSV was significantly reduced only in correspondence of vertebrae C1 and C2.

**Table 2:** Brain morphometric changes in AD patients.

	HC (*n* = 32) mean (SD)	AD (*n* = 28) mean (SD)	*p*-value
Brain structures (mm^3^)
ICV	1,573,086 (144439)	1,511,611 (139,532)	0.04*
WM	612,335 (11230)	540,237 (12,064)	<0.001*
GM	427,508 (6,492)	399,274 (6,975)	0.006*
RHip	3,602 (106)	2,932 (114)	<0.001*
LHip	3,591 (99)	2,822 (107)	<0.001*
LThal	7,013 (109)	6,433 (118)	0.001*
RThal	6,808 (109)	6,371 (117)	0.011*
LAmy	1,256 (41)	1,054 (44)	0.002*
RAmy	1,323 (63)	1,120 (66)	0.035*

Volumes of different brain structures expressed in mm^3^. Values are expressed as mean (SD). Significance was set at *p* = 0.05. *Refers to statistically significant values.

**Table 3:** Spinal cord morphometric changes in AD patients.

Vertebra	HC (*n* = 32) mean (SD)	AD (*n* = 28) mean (SD)	*p*-value
**Area (mm^2^)**
C1	69.8 (1.6)	63.1 (1.8)	0.009*
C2	65.7 (1.3)	60.2 (1.4)	0.008*
C3	62.5(1.4)	56.9 (1.6)	0.013*
C4	62.5 (1.6)	57.2 (1.7)	0.031*
C5	58.9 (1.6)	52.8 (1.7)	0.019*
C2-C3	65.1 (1.6)	58.3 (1.7)	0.007*
**Volume (mm^3^)**
C1	883.4 (27.3)	800.4 (29.3)	0.050*
C2	979.8 (28.4)	857.1 (30.6)	0.006*
C3	932.3 (29)	886.9 (31.2)	0.308
C4	882.3 (35.1)	807.9 (37.7)	0.168
C5	667 (34.5)	609.1 (37.1)	0.275
C2-C3	1,860.5 (66.8)	1,729.9 (71.7)	0.204

Cross-sectional area (in mm^2^) and volumes (in mm^3^) of spinal cord levels. Values are expressed as mean (SD). Significance was set at *p* = 0.05. *Refers to statistically significant values.

### AD Classification Based on Morphometric Data

**Figure 3:**
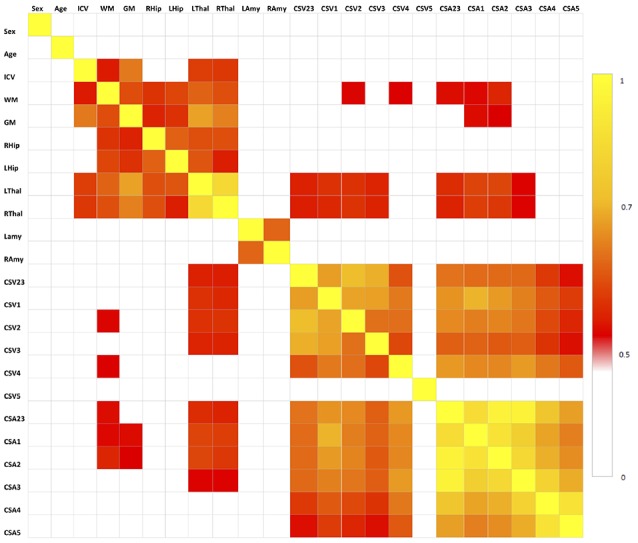
Correlation matrix between pair of variables tested with the Spearman’s correlation coefficient. All correlations for *p* < 0.5 are set to white, correlations for *p* > 0.5 are red to yellow, with yellow (*p* = 1) being the strongest correlation. No spinal cord metrics are correlating with brain metrics with *p* > 0.7, which is the threshold we used for extracting the set of uncorrelated features (Table 1).

The results of the correlation analysis are reported in Figure 3 and show that brain volumes are not significantly correlated with spinal cord metrics.

Features that were considered independent from each other and that were entered in the feature selection analysis are reported in Table 4. The best features selected by the RF algorithm for the AD vs. HC classification task are reported in Table 5 and include: RHip, WM, LAmy, LHip, CSA23, GM. Interestingly, CSA23 was identified as one of the most informative features to distinguish AD patients from HC. RF outcomes are reported in Table 6 and showed that the classification accuracy of AD patients is 76%, sensitivity 74%, and specificity 78%. The Area Under Curve (AUC) percentage reached 86%, showing a remarkable classification performance of the RF algorithm to distinguish AD from HC subjects. Moreover, it is noticeable that the hippocampi have dominant weight, but that there is a relevant contribution to the classification from CSA23.

**Table 4:** Cerebral and spinal cord morphometric metrics.

Set of all calculated metrics				Set of uncorrelated metrics			
Brain	Spine		Personal	Brain	Spine		Personal
WM	CSA1	CSV1	Age	WM	-	-	Age
GM	CSA2	CSV2	Gender	GM	-	-	Gender
RHip	CSA3	CSV3		LHip	-	CSV3	
LHip	CSA4	CSV4		RHip	-	-	
RThal	CSA5	CSV5		-	-	CSV5	
LThal	CSA23	CSV23		-	CSA23	-	
RAmy				-			
LAmy				LAmy			

*Left column: the initial dataset of morphometric metrics. Right column: a subset of uncorrelated morphometric metrics. WM, white matter; GM, gray matter; RHip, right hippocampus; LHip, left hippocampus; RThal, right thalamus; LThal, left thalamus; RAmy, right amygdala; LAmy, left amygdala; CSA, cross sectional area; CSV, cross sectional volume*.

**Table 5:** Features ranking.

Features	Weight
RHip	0.1125
WM	0.0630
LAmy	0.0629
LHip	0.0615
CSA23	0.0317
GM	−0.0041

*Nine HC and nine AD patients were used in the ranking procedure. Ranking Algorithm: ReliefF applied on a dedicated subset (30% of instances, number of neighbors = 10)*.

**Table 6:** Random forest classification.

Performance	
Accuracy	76%
Sensitivity	74%
Specificity	78%
Area under curve	86%
**Feature**	**RF weight**
LHip	9.039
RHip	2.734
LAmy	2.263
CSA23	1.828
WM	0.323
GM	0.060

*Twenty-three HC and 19 AD were used to test classifier performance. A leave-one-out procedure was used to test the performance of Random Forest (RF) with the best feature subset reported in Table 5. RF features weight are also reported*.

### MMSE and Morphometric Data Relationship

The combination of the six best features, including WM, RHip, LHip, LAmy, CSA23, and GM, explained 44% of the overall variance of the MMSE. The function equation describing the linear model obtained by the regression analysis included the following terms with their weights: 0.329*LHip-0.145*RHip+0.145*LAmy+0.064*CSA23-0.227*GM+0.557*WM. The MMSE explained variance was progressively reduced by simplifying the model, i.e., removing one or more predictors, as shown in Table 7. Each separate feature significantly (*p* < 0.005) explained a percentage of MMSE variance ranging between 13% and 36%. The feature that most explains MMSE variance was the WM volume (36%), with CSA explaining 13%.

**Table 7:** MMSE outcomes.

	Explained variance	Influence significance
**Multiple linear model**
MMSE = β_1_*LHip+β_2_*RHip+β_3_*LAmy+β_4*_CSA23+β_5_*GM+β_6_*WM	44%	<0.001
MMSE = β_1_*LHip+β_2_*RHip+β_3_*LAmy +β_4*_GM+β_5_*WM	43%	<0.001
MMSE = β_1_*LHip+β_2_*LAmy+β_3_*GM+β_4*_WM	43%	<0.001
MMSE = β_1_*LHip+β_2_*GM+β_3_*WM	42%	<0.001
MMSE = β_1_*LHip+β_2_*WM	40%	<0.001
**Linear model**	
MMSE = β*WM	36%	<0.001
MMSE = β*LHip	30%	<0.001
MMSE = β*RHip	22%	<0.001
MMSE = β*GM	17%	0.001
MMSE = β*LAmy	16%	0.001
MMSE = β*CSA23	13%	0.005

*MMSE Linear Regression Models. The model-explained variance is calculated with the R^2^ index. Significance was set to *p* = 0.05; all described model showed statistically significant influence (ANOVA)*.

## Discussion

The present work is pioneering the investigation of spinal cord alterations in patients with dementia, and in particular with AD, a major neurodegenerative disease known for its profound effects on cognitive functions. The motor/sensorimotor system has already been shown to be affected in AD at various levels in the brain, but nobody has yet investigated the spinal cord (Agosta et al., 2010; Salustri et al., 2013; Castellazzi et al., 2014; Albers et al., 2015; Fu et al., 2018). Here, we have shown that the spinal cord is significantly atrophic in established AD patients. This is an important finding, as it demonstrates that atrophy and neurodegeneration are widespread beyond areas with excellent standards such as the hippocampi and temporal lobes. Our results are, indeed, consistent with the fact that patients present significantly different brain volumes with respect to HC, and all segmented brain structures, except for the right amygdala, are statistically significantly atrophic in AD. In this context, our work goes further and demonstrates that volumes of all cervical vertebral segments are reduced in AD, with the CSV of the first and second vertebrae being significantly atrophic with respect to HC. These results are coherent with results obtained for cerebral structures and suggest the existence of a remarkable reduction (of the order of 10%) in the volume of the spinal cord in dementia. This hypothesis is further supported by significant CSA reduction for all vertebrae in patients, with CSA being calculated considering the curvature of the spinal cord (De Leener et al., 2017b). Previous studies have reported spinal cord atrophy in patients with neurological diseases (Okuda et al., 2014; Azodi et al., 2017), such as multiple sclerosis with focal lesions in the brain and spinal cord, but to date, no studies have explored the existence of a volumetric loss of spinal cord tissue in dementia.

This finding has implications on the way we should assess the cognitive and sensorimotor systems’ impairment, given that they are both affected in established AD, in order to highlight their possible relationship. Not to forget that the spinal cord is also the relay of the autonomic system that has been reported as dysfunctional in AD (Algotsson et al., 1995; Allan et al., 2007; Allan, 2019).

Post mortem studies of AD patients will be needed to confirm the biophysical source of spinal cord atrophy; although at first one could imagine that any change in CSA and CSV could be the result of retrograde Wallerian degeneration from the cerebral cortex (Alves et al., 2015), initial spinal cord post-mortem investigations of AD and HC has suggested that phosphorylates tau tangles are present especially in the cervical segment of the spinal cord of AD, even at the early stage (Dugger et al., 2013). One of the AD models (Yuan et al., 2013) showed that Aβ deposition in the spinal cord are visible 10 months after disease onset and predominantly selecting the corticospinal tract; they also show that such deposits are reduced when ablating the sensorimotor cortex, therefore suggesting that spinal cord Aβ deposits are secondary to terminal synaptic release. On the other hand, given that we have also demonstrated that spinal cord features are independent of brain volumes, it cannot be excluded that alterations in spinal cord morphometric measurements (CSA and CSV) in AD are the result of primary retrogenesis linked to myelin and axonal pathology. It is indeed very significant that a recent study of another animal model of AD (the 5xFAD) shows amyloid plaques accumulation in the spinal cord tissue, with a particular concentration at cervical level and a time-dependent accumulation that starts 11 weeks from onset; interestingly, the same study found independent and extensive myelopathy, while the motoneurons count at 6 months was not altered compared to the wild type (Chu et al., 2017). While, we cannot be conclusive on the mechanisms of spinal cord atrophy in AD, our results are intriguing and calling for larger studies of prodromic subjects to be followed over time; such studies would also confirm whether the suggestion that the motor system (neocortex, cerebellum and spinal cord) is affected even before the cognitive one can be substantiated, or whether the spinal cord is following similar pathophysiological global changes as brain structures (Agosta et al., 2010; Albers et al., 2015; Toniolo et al., 2018).

**Figure 4:**
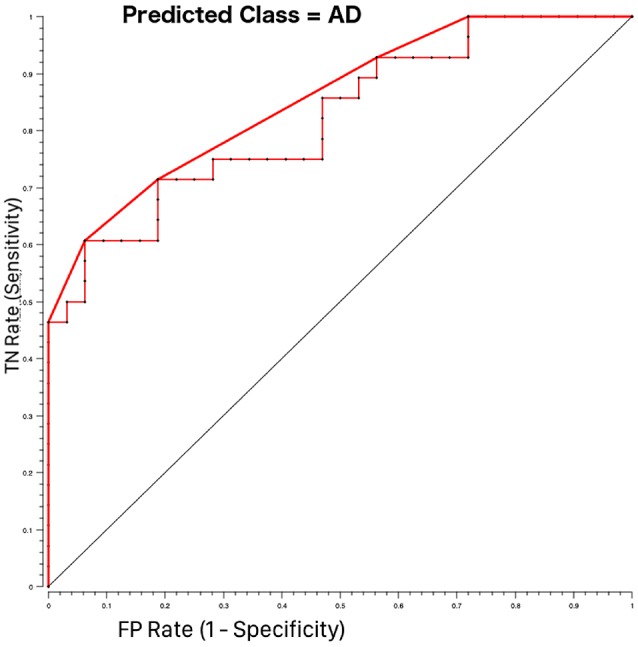
Receiving operating characteristics (ROC) curves for AD-HOC classification using Random Forest feature selection. The pathological class (AD = 1) was considered as the target class. The curve shows higher performance (bold red line) than the majority algorithm (diagonal). TN rate is the rate of true negatives and the FP rate is the rate of false positives.

A further result of our work is that of all spinal cord features analyzed here, the area of vertebra C2-C3 (CSA23) significantly contributes to discrimination between HC and AD patients. Usually, only atrophy of brain regions is investigated in dementia (Štepán-Buksakowska et al., 2014; Tardif et al., 2018). Indeed, spinal cord morphometric measures (CSA and CSV) alone cannot directly discriminate between AD and HC, but CSA23 was identified as one of the six best features useful to distinguish between these groups of subjects. Classifier accuracy was good and reached its best performance, around 76% when both volumes of brain structures, such as LHip and RHip [considered biomarkers of AD progression (O’Callaghan et al., 2019)], WM and GM, as well as spinal cord CSA23 were included in the classification procedure. In addition, the ROC curve between AD and HC (shown in Figure 4) reported high performance with AUC of 86%. The sensitivity and specificity of the RF algorithm, reaching 74% and 78%, respectively, showed a remarkable ability to correctly identify healthy and pathological cases. Examining the RF feature weighting (reported in Table 6) it is also noticeable that CSA23 had weight higher than GM, highlighting that it should be considered as an additional feature together with the more conventional volumes of subcortical regions (Pearson, 1900). These results indicate the yet unexplored potential influence that spinal cord features can play in the classification of dementia in line with recent publications, which have recognized that other brain structures play a key role in identifying AD patients and in distinguishing between different subtypes of dementia (Palesi et al., 2018; Ferreira et al., 2019).

Regarding the fact that CSA23 emerged as being particularly sensitive to pathological changes in AD is in accordance with other studies in neurodegenerative diseases such as amyotrophic lateral sclerosis (Antonescu et al., 2018) and could be seen as corroborating evidence of a correlation between spinal cord atrophy and neurodegeneration. In light of the only animal model study reported to date (Chu et al., 2017), which shows that C2-C3 is selectively affected by greater morphological biophysical alterations, our results become of remarkable value. Moreover, upper limb sensorimotor impairment is known to be clinically relevant in early AD, which is substantiating the relevance of our findings and calls for future investigations involving correlations with sensorimotor scores and purposely designed prospective studies to answer mechanistic questions.

Finally, our data show that also clinical aspects of AD are partially explained by spinal cord atrophy. Given the exploratory nature of the present study, we assessed whether spinal cord atrophy could be correlated with the variance of the MMSE, which is a global test, clinically used to assess AD severity. We found that 43% of the MMSE variance was explained with a multiple regression model implemented with all the best features included as independent variables, whereas CSA23 alone explained 13% of the MMSE variance, which is a considerable contribution indeed, after a 36% contribution of WM followed closely by 30% contribution of LHip. As a *post hoc* analysis, following these results, we assessed whether the contribution of WM atrophy and CSA23 to MMSE could be related to WM lesion burden. There was no correlation between these metrics and the Fazekas index (data not shown), which is a clinical measure that reflects vascular lesion load, therefore confirming that WM volume and CSA23 are most likely to reflect independent neurodegenerative mechanisms. Further studies should also confirm this finding.

From a methodological point of view, we know that evaluating spinal cord alterations in humans *in vivo* is challenging due to technical and anatomical constraints, including subject positioning inside the scanner, individual subject’s neck curvature or subject’s motion. Furthermore, the spinal cord is a small structure and optimized sequences with reduced FOV and appropriate alignment should be used to obtain reproducible results (De Leener et al., 2017a). Dedicated acquisition protocols would also allow one to analyze specific alterations of spinal cord GM and WM, that were not available with the present data that used 3DT1-w scans, used for whole-brain or regional brain volume calculations, to extract spinal cord features (Fonov et al., 2014; Levy et al., 2015). Regarding feature selection and classification, we know that recent studies have combined several MRI findings with machine learning approaches to attempt the classification of dementia subtypes and prediction of disease progression. Accuracy of about 80% (Amoroso et al., 2018; Waser et al., 2019) was achieved when AD and HC were classified while more fluctuating results were reported when more subtypes of dementia were considered. In the present study, an RF algorithm with the “leave-one-out” approach was chosen to discriminate between AD and HC because RF is robust with small numbers of subjects and performs features weighting runtime with good sensitivity and specificity.

Given the nature of this prospective study, it was not possible to investigate the involvement of the spinal cord at different stages of AD or in different types of dementia to explore its full clinical potentials. Therefore, a comprehensive battery of sensorimotor and cognitive tests should be performed to understand how the clinical and MRI pictures are evolving during the disease progression and to establish when spinal cord atrophy occurs and its clinical weight. It is also essential to promote multi-modal studies that can disentangle the contribution of myelin, amyloid accumulation, axonal swelling and axonal loss to brain and spinal cord alterations in neurodegenerative diseases to understand local and global mechanisms of damage.

In conclusion, the present work can be considered a milestone because for the first time in humans *in vivo* it demonstrates in a cohort of AD and HC subjects the contribution of spinal cord atrophy to explain clinical indicators of dementia and to improve disease classification, opening also mechanistic questions for future studies. It is indeed important that we rethink in particular of how the sensorimotor and cognitive systems are affected by AD, integrating spinal cord with brain information, including the temporal lobes with the hippocampi, the motor and sensorimotor cortices, the limbic system with the amygdala and the cerebellum, which we now know are all implicated in AD.

## Data Availability Statement

The datasets generated for this study are available on request to the corresponding author.

## Ethics Statement

The studies involving human participants were reviewed and approved by local ethic committee of the IRCCS Mondino Foundation. The patients/participants provided their written informed consent to participate in this study.

## Author Contributions

CG, ED’A, FP and RL conceptualized the study. FP and RL designed and performed the analyses with support from GC. PV and NA acquired all MRI data. ES and SB acquired all neuropsychological data helping for data interpretation. AC, ES, GM, and MC enrolled all patients and performed all clinical evaluations. CG and ED’A provided support and guidance with data interpretation with the clinical contribution of all physicians. CG, FP and RL wrote the manuscript, with comments from all other authors. This manuscript has been released as a Pre-Print at BioRxiv (Lorenzi et al., 2019).

## Conflict of Interest

The authors declare that the research was conducted in the absence of any commercial or financial relationships that could be construed as a potential conflict of interest.

## Footnotes

^1^https://github.com/neuropoly/spinalcordtoolbox

^2^https://www.fil.ion.ucl.ac.uk/spm/software/spm12

^3^https://fsl.fmrib.ox.ac.uk/fsl/fslwiki/FIRST

^4^https://orange.biolab.si/
